# Uncertainty Evaluations for Risk Assessment in Impact Injuries and Implications for Clinical Practice

**DOI:** 10.3389/fbioe.2020.00877

**Published:** 2020-08-07

**Authors:** Anjishnu Banerjee, Hoon Choi, Nicholas DeVogel, Yayun Xu, Narayan Yoganandan

**Affiliations:** ^1^Division of Biostatistics, Medical College of Wisconsin, Milwaukee, WI, United States; ^2^Center for NeuroTrauma Research, Department of Neurosurgery, Medical College of Wisconsin, Milwaukee, WI, United States

**Keywords:** confidence intervals, injury risk curves, survival analysis, NCIS quality measures, resampling

## Abstract

Injury risk curves (IRCs) represent the quantification of risk of adverse outcomes, such as a bone fracture, quantified by a biomechanical metric such as force or deflection. From a biomechanical perspective, they are crucial in crashworthiness studies to advance human safety. In clinical settings, they can be used as an assistive tool to aid in the decision-making process for surgical or conservative treatment. The estimation of risk corresponding to a level of biomechanical metric is done using a regression technique, such as a parametric survival regression model. As with any statistical procedure, error measures are computed for the IRC, representing the quality of the estimated risk. For example, confidence intervals (CIs) are recommended by the International Standards Organization, and the normalized confidence interval width (NCIW) is computed based on the width of the CI. This is a surrogate for the quality of the risk curve. A 95% CI means that if the same experiment were hypothetically repeated 100 times, at least 95 of the computed CIs should contain the true risk curve. Such an interpretation is problematic in most biomechanical contexts as rarely the same experiment is repeated. The notion that a wider confidence interval implies a poorer quality risk curve can be misleading. This article considers the evaluation of CIs and its implications in biomechanical settings for safety engineering and clinical practice. Alternatives are suggested for future studies.

## Introduction

Certain civilian and military scenarios induce traumatic loadings to the human body (falls and motor vehicle crashes in the former, and underbody blast from improvised explosive devices in the latter), and they may lead to injuries. The loading vector and mechanism of injuries may vary between scenarios. Despite the environmental differences between the two disciplines (younger/healthier versus older and age-related and or diseased populations, vehicle design differences, use of personal protective equipment (PPE) use such as helmets and body armor), priorities for the treatment and prevention/mitigation of injuries remain the same.

Clinicians use diagnostic images of the injuries such as x-rays and computed tomography for treatment: example, surgical options may depend on stability of the diseased/injured spine (Denis, 1983; Choi et al., 2019). Multiple segmented rib fractures may indicate the need for more aggressive chest injury treatment (Knopp et al., 1988; Cavanaugh et al., 1994; Yoganandan et al., 2013b). The goal of safety engineers is to reproduce field-observed injuries to develop standards for injury prevention/mitigation and improve vehicle designs, and clinical practice (FMVSS-208, 2001; FMVSS-214, 2008; Kutteruf et al., 2018). In both disciplines it is important to re-create field-observed injuries, understand their mechanisms, and determine human tolerance via robust statistical analysis. The former two are routinely accomplished via experiments with post-mortem human surrogates (PMHS) models.

The development of injury risk curves (IRCs) is a critical output from statistical analysis of PMHS data. The IRC estimation using a statistical technique such as survival regression is accompanied by estimation of confidence intervals (CIs) (Petitjean et al., 2012, 2015; Yoganandan and Banerjee, 2018). They are used to determine the normalized confidence interval width (NICW) to determine the quality of the IRC (Yoganandan et al., 2016a, 2017). In this article, the 95% CIs are used (Kuppa et al., 2003; Yoganandan et al., 2015).

The focus of this article is the evaluation of CIs for its potential use in safety engineering and medicine. It is appropriate to note the technical meaning of the confidence parameter and CI: CI means that if the same experiment that was used to construct the estimate and CI were to be repeated 100 times under the same underlying conditions, about 95 of those experiments should yield CIs which contain the true unknown IRC (Efron and Tibshirani, 1986). Impact biomechanical experiments are rarely repeated under exact conditions and the true IRC is always unknown. This poses challenges for statistical evaluation of the CIs. The objective of this study is to present a new methodology for evaluation of the CIs from an already generated impact biomechanical dataset and examine the coverage at different risk levels.

## Methods

### Application Dataset

A widely used biomechanical dataset from side impact sled tests was used in the study. There were 42 PMHS tests (Kuppa et al., 2003). Each PMHS specimen was subjected to side impact loading at different velocities, padding and rigid load wall conditions, offsets, and supplemental restraint systems (with and without side impact airbags). Each specimen was tested once. Injuries included unilateral or bilateral rib fractures in isolation or in combination with solid organ trauma. The presence and absence of injury were graded using the Abbreviated Injury Scale, and severities greater than 3 were classified as injurious (AIS, 1990). Biomechanical metrics included data from different types of sensors: accelerometers for the thoracic trauma index (TTI), peak pelvic and Average Spine Accelerations (ASA), and forces for the thoracic and pelvic regions from respective load cells (the reader is referred to the Table shown in the Appendix B, page 209, from the original publication).

Out of the 42 PMHS specimens, complete data were available for 37 tests. The reduced set of 37 complete experiments included 18 specimens with injury and 19 specimens without injury. This is termed as the evaluation dataset in this paper. The present analysis was performed for all biomechanical metrics, and results are reported for the 15 metrics that had the best Brier scores among the set of 33 metrics, indicating that they had the strongest association with injury outcome (Brier, 1950; Yoganandan and Banerjee, 2018; DeVogel et al., 2019).

### Overview of Coverage Estimation

A CI provides a plausible range of values for an unknown parameter. A 95% CI indicates that the chance the interval contains the true value of unknown parameter is 0.95. This means that if the experiment is repeated 100 times, approximately 95 of the CIs contain the true value of the unknown parameter. In the biomechanical context, it is not feasible to construct random repeated experiments for evaluating confidence intervals and the true IRC is unknown. Therefore, to evaluate the CIs, surrogate experiments using constrained resampling were used.

### Random Experiment Surrogates

The IRC computed using the evaluation dataset serves as the surrogate for the true unknown risk curve. The size of the evaluation subset is larger than what is typically observed for biomechanical studies, that usually have sample sizes in the range 10 to 30. To evaluate the performance of CI, 10,000 resampled subsets were created for each of the sample sizes, 10, 20, and 30. Each resampled subset represents an experimental surrogate for which a CI is computed. An estimate of how much of the surrogate IRC is contained within the CI interval was obtained and then averaged over the 10,000 replications for each sample size 10, 20, and 30. In effect, the 10,000 randomly sampled subsets, consisting of 10 samples each, spanned all datapoints from the evaluation dataset in a random order. The subsets spanning all datapoints in the evaluation dataset was also true for sample sizes 20 and 30.

### Constrained Resampling Techniques

A resampled subset is constructed by drawing, without replacement, a subsample of a given size from the evaluation dataset. The simplest way of constructing such a sample is by using simple random sampling without replacement (SRSWOR) (Chaudhuri and Adhikary, 1989). There are several difficulties in using a direct SRSWOR: it might lead to subsets of data being chosen with only injury or only no-injury observations, or with severe imbalance in favor of injury or no-injury observations. The imbalance could lead to non-convergence for some of the statistical algorithms, such as parametric survival regression, and/or lead to biased comparisons, since the application dataset of choice has approximately equal numbers of injury observations and no-injury observations (Kuppa et al., 2003).

### CI Construction

The IRC is estimated by using a parametric survival regression model. In general, this involves estimating the parameters of regression model and constructing the CI for all risk levels. To formalize notations, consider a biomechanical metric M, a parametric distribution function F with parameters α and β. The estimated risk for M can be represented as $\bar{r}=F\,\left(M;\bar{\alpha },\bar{\beta }\right)$, where $\bar{\alpha },\bar{\beta }$ are the estimated values of the parameters from the data. Standard software packages can be used for performing parametric survival analysis and computing a standard error estimate $\bar{r}$, denoted as se ($\bar{r}$). A naïve way of constructing an interval around the estimate $[\bar{r}$ would be to use ($\bar{r}$ ± critical value^∗^ se $[\bar{r}$]). However, since the likelihood function for the risk $\bar{r}$ estimation is not linear, the naïve interval would be inaccurate. More efficient intervals are obtained by using the delta method, where the interval is constructed on the linear scale and exponentiated using the delta method approximation. Owing to their superior statistical properties and their use in most recent IRC estimation, delta method intervals were used in this study (Yoganandan et al., 2014; Yoganandan and Banerjee, 2018).

### Evaluation Measures

Lower and upper tails: Frequently, the surrogate risk curve based on the evaluation set is not contained within the CI boundaries computed from the resampled datasets at the tails. So, evaluations of IRC containment within the CI boundaries were examined over three regions: (a) the whole IRC (between risk levels 0 to 1); (b) the lower tail defined as the portion of the IRC below risk level 0.33; and (c) the upper tail defined as the portion of the IRC above the 0.66 risk level.

Proportion and average length of coverage: For each of the three regions (overall curve, and lower and upper tails), the following measures were calculated: (a) the proportion, defined as the percent of times any portion of a region of interest from an estimated CI contained the surrogate risk curve, and (b) the average length, defined as the relative lengths of the region where the surrogate risk curve fell inside the estimated CI.

## Results

The average number of times resampled CIs contain the surrogate risk curve, for each of the metrics, and for each of the subset sizes is shown in Tables 1, 2, respectively. Table 1 shows the average length for the upper and lower tails and whole curve. Table 2 shows these data for the average proportion parameter.

**TABLE 1:** The average percentage of CI’s with any length containing the surrogate IRC, for the upper and lower tails and whole curve (all) for all three sample sizes IRC.

**Metric**	**Sample size**	**Coverage**	**Average length**	**Coverage**	**Average length**	**Coverage**	**Average length**
**ASA**	10	all	89.09	lower	85.71	upper	85.01
**fdavg**	10	all	88.35	lower	82.03	upper	90.49
**fdmax**	10	all	80.05	lower	69.41	upper	84.20
**fvavg**	10	all	79.83	lower	75.24	upper	75.98
**fvc**	10	all	88.68	lower	85.47	upper	84.32
**fvcavg**	10	all	88.46	lower	84.68	upper	85.23
**fvmax**	10	all	74.43	lower	70.25	upper	71.05
**hdavg**	10	all	86.26	lower	78.64	upper	90.02
**hdmax**	10	all	83.10	lower	74.24	upper	84.92
**hvavg**	10	all	85.52	lower	80.96	upper	81.15
**hvc**	10	all	88.17	lower	84.61	upper	84.50
**hvcavg**	10	all	87.17	lower	83.22	upper	82.70
**hvmax**	10	all	82.33	lower	77.35	upper	78.15
**pel**	10	all	83.45	lower	79.44	upper	78.86
**TTI**	10	all	90.38	lower	87.44	upper	87.93
**ASA**	20	all	96.30	lower	94.81	upper	94.65
**fdavg**	20	all	84.87	lower	74.57	upper	88.49
**fdmax**	20	all	77.52	lower	63.45	upper	81.74
**fvavg**	20	all	89.07	lower	85.71	upper	86.70
**fvc**	20	all	96.01	lower	94.32	upper	94.56
**fvcavg**	20	all	95.76	lower	93.72	upper	94.58
**fvmax**	20	all	83.22	lower	79.65	upper	80.76
**hdavg**	20	all	88.41	lower	78.66	upper	91.80
**hdmax**	20	all	86.02	lower	75.79	upper	88.32
**hvavg**	20	all	94.12	lower	91.75	upper	91.98
**hvc**	20	all	96.08	lower	94.24	upper	94.77
**hvcavg**	20	all	95.70	lower	93.92	upper	94.08
**hvmax**	20	all	91.00	lower	87.71	upper	88.65
**pel**	20	all	92.88	lower	90.59	upper	90.49
**TTI**	20	all	95.69	lower	94.01	upper	93.98
**ASA**	30	all	99.61	lower	99.42	upper	99.42
**fdavg**	30	all	91.64	lower	83.14	upper	95.69
**fdmax**	30	all	87.57	lower	80.29	upper	89.92
**fvavg**	30	all	97.79	lower	96.77	upper	97.31
**fvc**	30	all	99.64	lower	99.39	upper	99.57
**fvcavg**	30	all	99.56	lower	99.24	upper	99.49
**fvmax**	30	all	94.63	lower	92.86	upper	93.92
**hdavg**	30	all	93.95	lower	86.88	upper	96.94
**hdmax**	30	all	92.14	lower	84.29	upper	94.87
**hvavg**	30	all	99.28	lower	98.89	upper	99.02
**hvc**	30	all	99.57	lower	99.30	upper	99.45
**hvcavg**	30	all	99.53	lower	99.19	upper	99.43
**hvmax**	30	all	98.65	lower	97.92	upper	98.33
**pel**	30	all	98.82	lower	98.25	upper	98.35
**TTI**	30	all	99.25	lower	98.92	upper	98.87

*If the CI coverage were 95%, each of the numbers below should be approximately 95.*

**TABLE 2:** The average percentage of CI’s with any proportion containing the surrogate IRC, for the upper and lower tails and whole curve (all) for all three sample sizes IRC.

**Metric**	**Sample size**	**Coverage**	**Ave proportion**	**Coverage**	**Ave proportion**	**Coverage**	**Ave proportion**
**ASA**	10	all	80.64	lower	81.96	upper	81.46
**fdavg**	10	all	76.08	lower	77.67	upper	80.04
**fdmax**	10	all	63.06	lower	65.11	upper	66.65
**fvavg**	10	all	74.31	lower	74.40	upper	74.58
**fvc**	10	all	80.55	lower	82.08	upper	81.77
**fvcavg**	10	all	80.85	lower	81.85	upper	82.16
**fvmax**	10	all	69.90	lower	69.92	upper	70.16
**hdavg**	10	all	74.03	lower	74.68	upper	79.13
**hdmax**	10	all	68.95	lower	70.37	upper	72.63
**hvavg**	10	all	78.16	lower	78.73	upper	78.50
**hvc**	10	all	79.69	lower	81.67	upper	81.22
**hvcavg**	10	all	78.58	lower	79.66	upper	79.42
**hvmax**	10	all	75.83	lower	76.06	upper	76.16
**pel**	10	all	76.88	lower	77.52	upper	77.06
**TTI**	10	all	79.10	lower	82.58	upper	81.95
**ASA**	20	all	92.04	lower	92.68	upper	92.34
**fdavg**	20	all	70.50	lower	70.74	upper	74.43
**fdmax**	20	all	59.72	lower	60.07	upper	62.06
**fvavg**	20	all	85.08	lower	85.09	upper	85.39
**fvc**	20	all	92.42	lower	92.87	upper	92.90
**fvcavg**	20	all	91.99	lower	92.35	upper	92.60
**fvmax**	20	all	79.47	lower	79.47	upper	79.79
**hdavg**	20	all	72.97	lower	73.67	upper	77.98
**hdmax**	20	all	71.18	lower	71.66	upper	74.55
**hvavg**	20	all	89.97	lower	90.20	upper	90.22
**hvc**	20	all	92.16	lower	92.64	upper	92.58
**hvcavg**	20	all	91.66	lower	92.13	upper	92.08
**hvmax**	20	all	86.86	lower	86.90	upper	87.07
**pel**	20	all	89.01	lower	89.26	upper	89.14
**TTI**	20	all	88.69	lower	90.51	upper	89.81
**ASA**	30	all	98.87	lower	98.95	upper	98.88
**fdavg**	30	all	80.02	lower	80.04	upper	83.24
**fdmax**	30	all	80.26	lower	80.26	upper	80.26
**fvavg**	30	all	96.54	lower	96.54	upper	96.65
**fvc**	30	all	99.06	lower	99.10	upper	99.21
**fvcavg**	30	all	98.96	lower	98.98	upper	99.10
**fvmax**	30	all	92.77	lower	92.77	upper	93.17
**hdavg**	30	all	84.17	lower	84.19	upper	88.06
**hdmax**	30	all	81.94	lower	81.95	upper	84.73
**hvavg**	30	all	98.52	lower	98.57	upper	98.55
**hvc**	30	all	98.84	lower	98.87	upper	98.93
**hvcavg**	30	all	98.78	lower	98.81	upper	98.86
**hvmax**	30	all	97.69	lower	97.69	upper	97.81
**pel**	30	all	97.79	lower	97.84	upper	97.82
**TTI**	30	all	97.50	lower	97.92	upper	97.55

*If the CI coverage were 95%, each of the numbers below should be approximately 95.*

For the sample size of 10, for the lower tail, the proportion ranged from 65.11% (for the metric fdmax) to 82.58% (metric TTI); while the average length ranged from 69.41% (fdmax) to 87.43% (for TTI). For the upper tail, the proportion ranged from 66.65% (fdmax) to 82.16% (fvcavg); while the average length ranged from 71.05% (fvmax) to 90.49% (fdavg).

For the sample size of 20, for the lower tail, the proportion ranged from 60.07% (fdmax) to 92.87% (fvc); while the average length ranged from 63.45% (fdmax) to 94.81% (ASA). For the upper tail, the proportion ranged from from 62.06% (fdmax) to 92.9% (fvc); while the average length ranged from 80.76% (fvmax) to 94.7% (hvc).

For the sample size of 30, for the lower tail, the proportion ranged from 80.04% (for metric fdavg) to 99.1% (fvc); while the average length ranged from 80.29% (for metric fdmax) to 99.41% (ASA). For the upper tail, the proportion ranged from 80.26% (fdmax) to 99.21% (metric fvc); while the average length ranged from 89.92% (fdmax) to 99.57% (fvc).

## Discussion

The present analysis used a representative dataset from a large body of tests conducted in our laboratory (Kuppa et al., 2003). While it was focused on skeletal injuries to the thoracic rib cage, the same type of analysis can be used for spine trauma. Spine trauma studies have developed IRCs for injuries such as cervical and thoracolumbar fractures (Yoganandan et al., 2013a,c, 2015, 2016b, 2018). It should be noted that statistical significance does not always imply or equate to clinical significance. For example, differences in force between two types of injuries (wedge fracture without instability or ligament laxity, versus wedge fracture with instability) may not be statistically significant, while the latter type of fracture often require surgical intervention (Maiman and Yoganandan, 1991; Maiman et al., 2002). CIs in such cases may need additional consideration. Clinically or scientifically meaningful differences should be considered in conjunction with confidence intervals.

### Uncertainty Quantification

Uncertainty quantification is an essential part of any statistical procedure. If IRCs are to be used as a predictive tool for clinical decision making it would be important to know the reliability of the estimates (Khor et al., 2018). Similar perspectives hold true in the applications of IRCs in biomechanical engineering for advancing human safety. This article demonstrates that standard CI construction as currently used, is deficient in the representation of this uncertainty. Some alternatives are suggested in subsection, “Alternative Methods for Interval Construction.”

### Coverage of an Interval

Typically, for any statistical method proposed or for a new learning paradigm, researchers investigate the actual versus the theoretical coverage using simulated data. With simulated data, as the generation mechanism is completely known to the experimenter beforehand, such coverage evaluations do not require the construction of resampled datasets. However, in the context of IRCs, constructing simulated datasets is difficult because they do not accurately mimic the exact variability associated with biomechanical experimental data.

For confidence interval evaluations, surrogates have been created using constrained SRSWOR subsets, to mimic experiments with smaller sample sizes. However, these subsets are not independent. With strict statistical interpretation, the CI should be evaluated against independent experiments under the same experimental conditions. However, it should be noted that completely independent repeatable experiments may lead to more varied IRCs, which in turn may lead to poorer coverage.

### Quality Implications

One of the crucial uses of the CI in the IRC context is its use in the estimation of the NCIW measures at different levels, as they serve as quality indicators for IRC’s, often governing the decision for adopting an IRC (Petitjean et al., 2015). The present study raises questions on the accuracy and validity of the CI, particularly for lower (<0.33) and higher (>0.66) risk levels. Based on the results of this study, alternative methods of quality measurements and benchmarking ought to be considered for IRCs.

### Alternative Methods for Interval Construction

Several alternative modes of confidence interval construction exist, as applied to the IRC context. Resampling tools which were used for evaluation, could be used for non-parametric confidence interval construction, even if the underlying risk curve estimation is from a parametric survival regression (Efron and Tibshirani, 1986). However, these would be difficult to implement in low sample size settings. Non-parametric intervals have been known to be less efficient but more flexible than parametric CI. Likelihood-based alternatives may include Bayesian methods: a prior is constructed based on past experiments and the standard parametric survival regression likelihoods are used in conjunction with the priors to yield Bayesian credible intervals (Ibrahim et al., 2001). One of the major advantages of a Bayesian credible interval is that it can be interpreted in probabilistic terms (Yoganandan et al., 2020). A 95% Bayesian credible interval would be interpreted more directly and simply as the interval such that the probability of an estimate belonging to this interval is 95%, as opposed to the repeated experiment interpretation of classical frequentist confidence intervals (Ibrahim et al., 2001). The difficulty with the Bayesian credible intervals is the sensitivity to the prior. For low sample settings, the prior could have large influence on the estimates (Martz, 2014). These approaches should be considered in future studies as better scientific representations of the biomechanical uncertainty for IRCs that may be used in safety engineering and clinical practice for standard of care.

### Limitations and Strengths

The present study adopted a widely used experimental dataset to meet the objectives of the study. The specific results shown in Tables 1, 2 are, therefore, applicable to only the evaluation dataset that consisted of 15 metrics from a limited set of 37 experiments. However, the data set of 37 samples is relatively large for an injury biomechanics data set. The underlying assumption was that the 5 unused tests did not capture the 15 metrics that were ultimately chosen for their strong association with injury outcome. Furthermore, the parametric survival analysis treated injury data as left censored, and this was because the timing of the injuries was not known from the experiments. As uncensored treatment adds certainty to the datapoint/observation, the IRCs tended to shift rightwards, i.e., greater biomechanical metric (e.g., force) associated with a specific injury risk. This may change the uncertainty coverage; however, the SRSWOR can still be used with the constraint of matching the ratio of injury to non-injury datapoints. As each specimen was tested once, data were right or left censored, depending on the non-injury or injury status. In cases where repeated testing is done resulting in non-injury and injury datapoints for the same specimen, interval censoring should be used. It should be noted that as the number of datapoints in the resampled dataset approaches the evaluation dataset, the randomization effect reduces. This may result in an overestimation of the CI coverage. The present study showing that the CI coverage is non-uniform across risk levels of IRCs is new to the impact and injury biomechanics field. The strengths of the study include the detailed evaluation of confidence intervals which have not been adequately interrogated in this context before. Another strength is that the current method does not use simulated data.

### Summary

Using a large impact biomechanics dataset, the SRSWOR method was used to show that the proportion and average length of coverage increase with sample size; however, most of the coverage for both parameters were below 95%. In general, the coverage was non-uniform across all risk levels. In addition, the performance of the CI varied for different metrics, representing different sensitivities of each metric for the injury outcome.

## Data Availability Statement

All datasets generated for this study are included in the article/supplementary material/available from the original paper cited in this article (Kuppa et al., 2003).

## Author Contributions

AB, ND, and YX contributed to the design and statistical analysis. NY and HC contributed to the write-up, scientific perspectives, review of the completed draft, and accuracy. All authors contributed to the article and approved the submitted version.

## Conflict of Interest

The authors declare that the research was conducted in the absence of any commercial or financial relationships that could be construed as a potential conflict of interest.

## Abbreviations

fdavg: Average full thorax deflection
hdavg: Average half thorax deflection
ASA: Average Spine Acceleration
fvavg: Average velocity derived from full thorax deflection
hvavg: Average velocity derived from half thorax deflection
fvcavg: Average viscous criterion derived from full thorax deflection
hvcavg: Average viscous criterion derived from half thorax deflection
CI: confidence interval
IRC: injury risk curve
fdmax: Maximum full thoracic deflection
hdmax: Maximum half thoracic deflection
fvmax: Maximum velocity derived from full thorax deflection
hvmax: Maximum velocity derived from half thorax deflection
fvc: Maximum viscous criterion derived from full thorax deflection
hvc: Maximum viscous criterion derived from half thorax deflection
NCIW: Normalized Confidence Interval Width
pel: Pelvic wall force
PMHS: Post-Mortem Human Subjects
SRSWOR: Simple Random Sampling Without Replacement
TTI: Thoracic Trauma Index.

## References

[B1] AIS (1990). *The Abbreviated Injury Scale, 1998 Update.* Arlington Heights, IL: American Association for Automotive Medicine.

[B2] Brier G. W. (1950). Verification of forecasts expressed in terms of probability. *Month. Weather Rev.* 78 1–3. 10.1175/1520-0493(1950)078<0001:vofeit>2.0.co;2

[B3] Cavanaugh J. Zhu Y. Huang Y. King A. I. (1994). “Injury and response of the thorax in side impact cadaveric tests,” in *Biomechanics of Impact Injury and Injury Tolerances of the Thorax-Shoulder Complex*, ed. Backaitis S. (Warrendale, PA: SAE, Inc), 949–972.

[B4] Chaudhuri A. Adhikary A. K. (1989). On efficiency of the ratio estimator. *Metrika* 36 55–59. 10.1007/bf02614078

[B5] Choi H. Baisden J. L. Yoganandan N. (2019). A comparative in vivo study of semi-constrained and unconstrained cervical artificial disc prostheses. *Mil. Med.* 184(Suppl. 1), 637–643. 10.1093/milmed/usy395 30901460

[B6] Denis F. (1983). The three column spine and its significance in the classification of acute thoracolumbar spinal injuries. *Spine* 8 817–831. 10.1097/00007632-198311000-00003 6670016

[B7] DeVogel N. Banerjee A. Yoganandan N. (2019). Application of resampling techniques to improve the quality of survival analysis risk curves for human frontal bone fracture. *Clin. Biomech.* 64 28–34. 10.1016/j.clinbiomech.2018.04.013 29753560

[B8] Efron B. Tibshirani R. (1986). Bootstrap methods for standard errors, confidence intervals, and other measures of statistical accuracy. *Statist. Sci.* 77 54–75. 10.1214/ss/1177013815

[B9] FMVSS-208 (2001). *Federal Motor Vehicle Safety Standards.* Washington, DC: US Government.

[B10] FMVSS-214 (2008). *Federal Motor Vehicle Safety Standards.* Washington, DC: US Government.

[B11] Ibrahim J. G. Chen M. H. Sinha D. (2001). *Bayesian Survival Analysis.* Cham: Springer Science & Business Media.

[B12] Khor S. Lavallee D. Cizik A. M. Bellabarba C. Chapman J. R. Howe C. R. (2018). Development and validation of a prediction model for pain and functional outcomes after lumbar spine surgery. *JAMA Surg.* 153 634–642. 29516096 10.1001/jamasurg.2018.0072 PMC5875305

[B13] Knopp R. Yanagi A. Kallsen G. Geide A. Doehring L. (1988). Mechanism of injury and anatomic injury as criteria for prehospital trauma triage. *Ann. Emerg. Med.* 17 895–902. 10.1016/s0196-0644(88)80666-8 3415061

[B14] Kuppa S. Eppinger R. H. McKoy F. Nguyen T. Pintar F. A. Yoganandan N. (2003). Development of side impact thoracic injury criteria and their application to the modified es-2 dummy with rib extensions (ES-2re). *Stapp. Car. Crash. J.* 47 189–210. 17096250 10.4271/2003-22-0010

[B15] Kutteruf R. Wells D. Stephens L. Posner K. L. Lee L. A. Domino K. B. (2018). Injury and liability associated with spine surgery. *J. Neurosurg. Anesthesiol.* 30 156–162. 10.1097/ana.0000000000000448 28763433

[B16] Maiman D. J. Yoganandan N. (1991). Biomechanics of cervical spine trauma. *Clin. Neurosurg.* 37 543–570. 2009707

[B17] Maiman D. J. Yoganandan N. Pintar F. A. (2002). Preinjury cervical alignment affecting spinal trauma. *J. Neurosurg.* 97(1 Suppl.), 57–62. 10.3171/spi.2002.97.1.0057 12120652

[B18] Martz H. F. (2014). *Bayesian Reliability Analysis. Wiley StatsRef.* Available online at: https://onlinelibrary.wiley.com/doi/abs/10.1002/9781118445112.stat03957 (accessed June 7, 2020).

[B19] Petitjean A. Torsseille X. Yoganandan N. Pintar F. A. (2015). “Normalization and scaling for human response corridors and development of risk curves,” in *Accidental Injury: Biomechanics and Prevention*, eds Yoganandan N. Nahum A. M. Melvin J. W. (New York, NY: Springer), 769–792. 10.1007/978-1-4939-1732-7_26

[B20] Petitjean A. Trosseille X. Praxl N. Hynd D. Irwin A. (2012). Injury risk curves for the WorldSID 50th male dummy. *Stapp. Car. Crash. J.* 56 323–347. 23625565 10.4271/2009-22-0016

[B21] Yoganandan N. Arun M. W. Pintar F. A. Szabo A. (2014). Optimized lower leg injury probability curves from postmortem human subject tests under axial impacts. *Traffic Inj. Prev.* 15(Suppl. 1), S151–S156. 25307381 10.1080/15389588.2014.935357 PMC4430105

[B22] Yoganandan N. Arun M. W. Stemper B. D. Pintar F. A. Maiman D. J. (2013a). Biomechanics of human thoracolumbar spinal column trauma from vertical impact loading. *Ann. Adv. Automot. Med.* 57 155–166. 24406955 PMC3861829

[B23] Yoganandan N. Pintar F. A. Humm J. R. Stadter G. W. Curry W. H. Brasel K. J. (2013b). Comparison of AIS 1990 update 98 versus AIS 2005 for describing PMHS injuries in lateral and oblique sled tests. *Ann. Adv. Automot. Med.* 57 197–208. 24406958 PMC3861828

[B24] Yoganandan N. Stemper B. D. Pintar F. A. Maiman D. J. McEntire B. J. Chancey V. C. (2013c). Cervical spine injury biomechanics: applications for under body blast loadings in military environments. *Clin. Biomech.* 28 602–609. 10.1016/j.clinbiomech.2013.05.007 23796847

[B25] Yoganandan N. Banerjee A. (2018). Survival analysis-based human head injury risk curves: focus on skull fracture. *J. Neurotrauma* 35 1272–1279. 10.1089/neu.2017.5356 29409390

[B26] Yoganandan N. Chirvi S. Pintar F. A. Uppal H. Schlick M. Banerjee A. (2016a). Foot-ankle fractures and injury probability curves from post-mortem human surrogate tests. *Ann. Biomed. Eng.* 44 2937–2947. 10.1007/s10439-016-1598-2 27052746

[B27] Yoganandan N. Pintar F. A. Humm J. R. Maiman D. J. Voo L. Merkle A. (2016b). Cervical spine injuries, mechanisms, stability and AIS scores from vertical loading applied to military environments. *Eur. Spine J.* 25 2193–2201. 10.1007/s00586-016-4536-y 27043728

[B28] Yoganandan N. DeVogel N. Pintar F. Banerjee A. (2020). Human pelvis bayesian injury probability curves from whole body lateral impact experiments. *J. Eng. Sci. Med. Diagn. Ther.* 3:031002. 10.1115/1.4046672 PMC8597554 35832784

[B29] Yoganandan N. Moore J. Pintar F. A. Banerjee A. DeVogel N. Zhang J. (2018). Role of disc area and trabecular bone density on lumbar spinal column fracture risk curves under vertical impact. *J. Biomech.* 72 90–98. 10.1016/j.jbiomech.2018.02.030 29559244

[B30] Yoganandan N. Nahum A. N. Melvin J. W. (2015). *Accidental Injury: Biomechanics and Prevention.* New York, NY: Springer.

[B31] Yoganandan N. Pintar F. A. Banerjee A. (2017). Load-based lower neck injury criteria for females from rear impact from cadaver experiments. *Ann. Biomed. Eng.* 45 1194–1203. 10.1007/s10439-016-1773-5 28091968

